# Effect of Low Atmospheric Pressure on Air Entrainment in Cement-Based Materials: An On-Site Experimental Study at Different Elevations

**DOI:** 10.3390/ma13183975

**Published:** 2020-09-08

**Authors:** Xin Chen, Xu Liu, Bo Tian, Yong Ge, Lihui Li

**Affiliations:** 1 School of Transportation Science and Engineering, Harbin Institute of Technology, Harbin 150090, China; xin.chen@alu.hit.edu.cn (X.C.); 20b932012@stu.hit.edu.cn (X.L.); 2 Research Institute of Highway, Ministry of Transport, Beijing 100088, China; b.tian@rioh.cn

**Keywords:** concrete, mortar, cement paste, air content, air-entraining agent, low atmospheric pressure, high elevation, temperature, on-site study, Boyle-Mariotte’s law

## Abstract

The efficiency and stability of air entrainment in concrete are sometimes found to be weaker at higher elevation. This phenomenon was attributed to the low atmospheric pressure by many researchers, however, the level of influence of atmospheric pressure on concrete air content dramatically varied among different studies. In order to clarify the effect of low atmospheric pressure on air entrainment in cement-based materials, an on-site experimental study was conducted with a rigorous control of irrelevant variables. The study focused on the air-entraining efficiency in cement paste, mortar, and concrete prepared in both low and standard atmospheric pressures. The air bubble stability in fresh mortar and air void characteristics of hardened mortar in different atmospheric pressures were also included. In the study, little effect of low atmospheric pressure on the air-entraining efficiency and air bubble stability in mortar with studied air-entraining agents (AEAs) was found. The air void characteristics were found to be similar between mortar with SJ-2 or 303R type AEAs prepared in different atmospheric pressures. Concrete with either SJ-2 or 303R type AEA prepared in low atmospheric pressure presented a satisfactory air content. These conclusions indicate that it is not necessary to worry excessively about the potentially adverse effect of atmospheric pressure on the frost resistance of concrete if a suitable AEA is applied. Additionally, a supplementary mortar study found that the low temperature of raw materials stored at high elevation would significantly weaken the air entrainment, reminding that potential causes in addition to low atmospheric pressure should also be taken seriously.

## 1. Introduction

Concrete durability at high elevation has attracted the attentions of increasing researchers [1,2,3,4]. At high elevations, climate features including low atmospheric pressure, low relative humidity, low average temperature, and large range of diurnal temperature cycles are not friendly for concrete structures. Low average temperature coupling with a large range of diurnal temperature cycles delivers more annual freeze-thaw cycles over there [4,5]. The most effective way for concrete to improve frost resistance is increasing its air content by air-entraining agent (AEA). However, in engineering practice, some researchers and engineers found that the air content of fresh concrete in the Qinghai-Tibet Plateau, which is known as the roof of the world, was lower than that in low elevation regions. In their opinion, this phenomenon is owing to the atmospheric pressure difference [6,7]. To prove this opinion, many works have been done, which mainly include the following: (i) shaking/agitation of AEA solutions in different air pressures [5,8,9]; (ii) concrete air content test at different elevations, corresponding to different atmospheric pressures [3,4,5,9,10,11]; and (iii) air content test of concrete mixed in different simulated air pressures [12,13,14,15,16]. The majority of these works [9,10,12,13,14,15,16] drew a common conclusion, stating that low atmospheric pressure is the main cause of low air content of concrete in the Qinghai-Tibet Plateau. However, there were huge differences between what different people claimed about how much the air content of concrete would be affected. Moreover, many of these works were not designed considerately and some of them even violated usual physics laws.

To study the effect of atmospheric pressure on the foaming properties of AEA, some researchers manually shook a glass tube that contained certain AEA solution at different elevations [5,9]. They found that the foam columns were lower and declining faster in Qinghai compared with those in Beijing or Xi’an. This means that both foaming capacity and foam stability of AEAs at higher elevation are weaker. However, this conclusion became debatable recently. Li [8] conducted a similar test, in which he applied a mechanical agitation at a much higher speed. He found that neither the foam generation nor foam development in AEA solution was affected by the air pressure. Moreover, it is easy to understand that bubbles of solution cannot perfectly match bubbles in concrete:

– The nature of a bubble in the glass tube is a layer of spherical liquid film, which has two curved surfaces (outer liquid-inner gas surface and outer gas-inner liquid surface), while the air bubble in fresh concrete has only one curved surface (outer liquid-inner gas surface).
– In the glass tube, there is a gas-liquid two-phase system, while in fresh concrete there is a gas-liquid-solid (bubble-paste-aggregate) three-phase system.
– Bubbles observed and analyzed in the glass tube are at the top of liquid phase, however, in fresh concrete, bubbles that emerge to the surface would vanish quickly.

Therefore, the best way to study AEA properties in low atmospheric pressure is still concrete test, however, variables irrelevant to the atmospheric pressure should be strictly controlled.

Mix proportion, cementitious materials, and chemical admixtures were controlled in nearly all on-site studies [3,4,5,9,10,11], but only a few studies [3,4] controlled the aggregate sources. Moreover, few publications mentioned that the temperature of raw materials, instead of the temperature of the laboratory only, was kept the same when tests were conducted at different elevations. These two variables might induce significant errors to the research results. Firstly, a high clay content, which would negatively affect air entrainment, is a typical concern of aggregate quality in the Qinghai-Tibet Plateau [5]. Secondly, raw materials stored at high elevation are much colder than those stored at low elevation. In some projects in the Qinghai-Tibet Plateau, the mixing water was drawn from local rivers [5], which are the snowmelt from mountains. The highest temperature of the river water in the hottest summer is only 8–10 °C [5]. The cold temperature of raw materials is an important factor that reduces the air content of concrete.

For the considerations of altitude stress risks, logistical and experimental conditions, irrelevant variables control, as well as travelling time and expense, some researchers studied the effect of atmospheric pressure on air content of air-entrained concrete by simulating different air pressures in a laboratory instead of conducting tests at different elevations [12,13,14,15,16]. Li and Fu [12,13,14] firstly applied this methodology in their works, which strongly influenced later researchers [15,16], whose methodologies were the same as that applied by Li and Fu [12,13,14]. By the methodology, concrete is mixed in a sealed box with simulated air pressures, however, the simulated air pressures have to recover to local atmospheric pressure when mixing finishes, as, only if the concrete is taken out from the sealed box, can it experience subsequent tests. According to the Boyle-Mariotte’s law (P_1_V_1_ = P_2_V_2_), air bubbles entrained in low air pressure during mixing would be inevitably compressed when the ambient pressure rises to standard atmospheric pressure after mixing finishes. Unfortunately, these researchers [12,13,14,15,16] ignored the compression by citing a paper of Ley [17], which stated that the shell damage of air bubbles with a diameter less than 200 μm was difficult to observe in 500× magnification. The description from Ley [17] was fine, but obviously, it was misinterpreted by theses researchers as that small air bubbles are not compressible. The ignorance of the compressibility of air bubbles inside concrete indicates a negation of the Boyle-Mariotte’s law, which is exactly the operational principle of air content measurement by the pressure method [18,19]. If the air bubbles inside concrete are incompressible, then the needle on the dial of pressure gauge would not move, which means the air content would never be detected. Paradoxically, these researchers still applied the pressure gauge method to test the air content of air-entrained concrete [12,13,14,15,16]. A critical discussion about the outcomes of these studies is provided in Appendix A.

This paper delivers a comprehensive study on air entrainment efficiency and stability in cement-based materials in both low and standard atmospheric pressures. Given the technical barrier that concrete cannot be tested in a sealed environment, the study was conducted on-site at different elevations instead of basing on simulated pressures. For tests on cement paste and mortar, specimens were prepared in Shigatse (3860 m, 64 kPa) and Harbin (150 m, 101 kPa), respectively. In these tests, the density of fresh cement paste, the air content of mortar at both fresh and hardened states, the air bubble stability in fresh mortar, and the air void characteristics of hardened mortar were investigated. However, concrete specimens for air content tests were prepared only in Lhasa (3646 m, 66 kPa) instead of at different elevations, because coarse aggregates were difficult to keep the same in two places separated by great distance. The study found that cement paste density, mortar air content, and air bubble stability in mortar were not significantly affected by atmospheric pressure change. Moreover, the air void characteristics of hardened mortar with SJ-2 or 303R type AEA were similar at either atmospheric pressures. Concrete with either SJ-2 or 303R type AEA, prepared in low atmospheric pressure, presented a satisfactory air content. Additionally, it is found that the low temperature of raw materials stored at high elevation should be an important factor causing poor air entrainment. These conclusions dispel excessive worries about the potentially negative effect of low atmospheric pressure on air entrainment in cement-based materials and remind one that people pay more attention to other factors that may adversely affect air entrainment at high elevation.

## 2. Materials and Methods 

### 2.1. Test Site

Laboratories that supported the on-site study are respectively located in the following three cites:

– the Shigatse Branch of Sichuan Zhentong Highway Test Consultants Co., Ltd., Shigatse, Tibet;
– School of Transportation Science and Engineering, Harbin Institute of Technology, Harbin, Heilongjiang;
– the High and Cold Area and High Altitude Region’s Highway Engineering Materials Technology Key Laboratory, Transportation Survey, Design and Research Institute of Tibet Autonomous Region, Lhasa, Tibet.

The study was conducted in December 2019. The environmental conditions during the study are shown in Table 1 and Table 2.

### 2.2. Raw Materials

#### 2.2.1. Raw Materials Selection

All cement used in the study was reference cement (P·I 42.5 Portland cement) bought from Qufu Branch of China United Cement Corporation Co., Ltd (Qufu, China). This cement is exclusively used to test the properties of chemical admixtures for concrete according to GB8076-2008 [20].

All fine aggregates used in the study were ISO standard sand produced by China National Building Materials Croup Co., Ltd. (Xiamen, China), which is the only standard sand supplier in Chinese Mainland.

As only the concrete test in Lhasa needed coarse aggregates, coarse aggregates were granitic pebbles from the Brahmaputra River in Tibet. The diameter range of the pebbles was 5–25 mm.

Mixing water used in the study was local tap water, the temperatures of which are shown in Table 2.

Three types of AEAs were applied in the study. The AEA information is shown in Table 3.

#### 2.2.2. Raw Materials Pretreatments

Cement and aggregates were stored outdoors in Lhasa and Shigatse, but indoors in Harbin. The storage temperature can be found in Table 1 and Table 2. In order to control variables irrelevant to atmospheric pressure, raw materials needed to experience some pretreatments; see Table 4.

Exceptionally, a supplementary mortar test conducted in Harbin used raw materials without any pretreatment, because the aim of it was to investigate the effect of the temperature of raw materials on the air content. Temperatures of fresh mortar with and without pretreatments were 10 ± 1 °C and 20 ± 1 °C, respectively.

#### 2.2.3. Mix Proportions

The study covered tests for cement paste, mortar, and concrete. Mix proportions are shown in Table 5. In air-entrained cement-based materials, dosages of AEA (AEA/cement) are shown in Table 3.

Cement-based materials in the study were mixed in accordance with JEG E30-2005 [21]. All operations as well as the equipment types were kept the same when mortar or cement paste was prepared in different laboratories (moulds were stored at laboratory temperatures). The strength grade of concrete without air entrainment was C30.

### 2.3. Test Methods

#### 2.3.1. Density of Cement Paste

Although the concept of air content is not used for cement paste, the density of fresh air-entrained cement paste was studied to reflect the porosity difference between air-entrained cement paste prepared in different atmospheric pressures. The density of fresh cement paste was tested by an Erlenmeyer flask with a known capacity.

#### 2.3.2. Air Content and Air Bubble Stability of Mortar

Air content of fresh mortar was tested in accordance with Method A—Pressure method described in BS EN 1015-7:1999 [18]. There were two testing times in the study, one of which was the finish of mixing, while the other was 45 min after the mixing finished. It should be noted that each batch of fresh mortar could only experience one test instead of two, otherwise the accuracy of the second test would be affected. The air content difference between mortar tested in different times indicated the stability of entrained air bubbles.

For hardened mortar tests, fresh mortar was placed in moulds after a 45 min rest and then cured to 28 d age. Air content of hardened mortar at 28 d age was tested by the density method. The density of mortar was tested in saturated surface dry condition, in accordance with BS EN 12390-7:2009 [22]. Assuming that the air content of no-air-entrained mortar was constant at both fresh and hardened states, the air content of hardened air-entrained mortar can be calculated through Equation (1).

(1) $$A_{\mathrm{AEA}}=100-\frac{\rho_{\mathrm{AEA}}\;}{\;\;\rho_{0}}\left(100-A_{0}\right),$$

where

*A*_AEA_—the air content of air-entrained mortar at hardened state, %;

*A*_0_—the air content of no-air-entrained mortar at fresh state, %;

*ρ*_AEA_—the density of air-entrained mortar at hardened state, kg/m^3^;

*ρ*_0_—the density of no-air entrained mortar at hardened state, kg/m^3^.

#### 2.3.3. Air Content of Fresh Concrete

Air content of fresh concrete was tested by pressure gauge method, in accordance with BS EN 12350-7:2000 [19].

#### 2.3.4. Air Void Characteristics of Hardened Mortar

Cross sections cut from hardened mortar cubes (70.7 mm × 70.7 mm) were used to conduct air void characteristics analysis. Before the test, cross sections were brushed into black and exposed air voids filled with white powders. The test was conducted automatically by an air void spacing factor analyzer NELD-BS610, produced by Peking Neld Intelligen Co., Ltd. (Beijing, China). The test results given by the analyzer included air content, average radius, specific area, spacing factor, and so on.

## 3. Results

### 3.1. Densities of Fresh Cement Paste Prepared in Different Atmospheric Pressures

**Figure 1:**
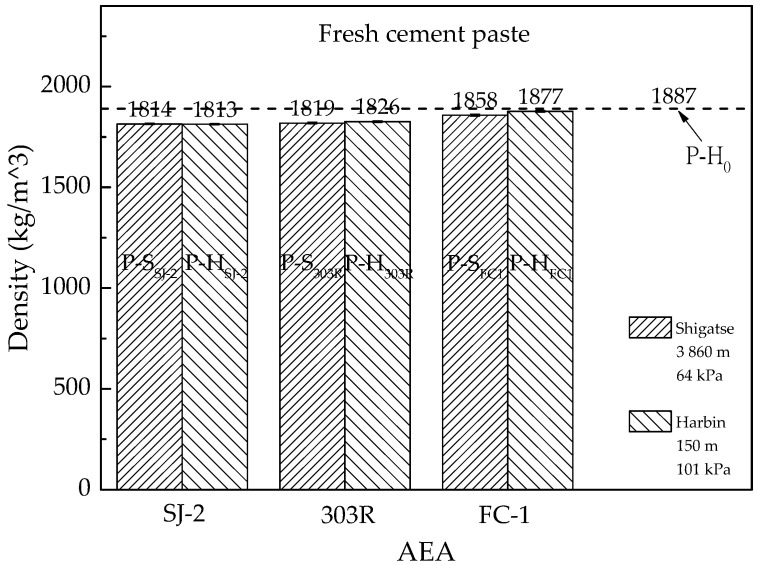
Density comparison of fresh air-entrained cement paste prepared in different atmospheric pressures. AEA, air-entraining agent.

The density comparison of fresh air-entrained cement paste prepared in different atmospheric pressures is shown in Figure 1. Each result is the average of three tests, with 300 mL fresh cement paste per test.

As air bubbles were entrained in the cement paste, the density of cement paste reduced. Densities of fresh cement paste with SJ-2 type AEA mixed in low and standard atmospheric pressures were very similar to each other. However, fresh cement paste with either 303R or FC1 type AEA performed better air entrainment in low atmospheric pressure compared with standard atmospheric pressure.

In Figure 1, the density reduction of air-entrained cement paste compared with no-air-entrained cement paste P-H_0_ was at a range of 0.5%–3.9%. The limited air entrainment in cement paste was owing to the lack of fine aggregates, which are important to improve the cohesiveness of fresh mixtures. Air bubbles in low viscosity fluid would be easy to emerge and escape, thus fresh cement paste cannot detain much air content inside like fresh mortar and concrete. Therefore, it is not feasible to use cement paste in an air entrainment study to predict mortar or concrete properties.

### 3.2. Air Entrainment Efficiency and Stability in Mortar Prepared in Different Atmosphric Pressures

#### 3.2.1. Fresh Mortar

**Figure 2:**
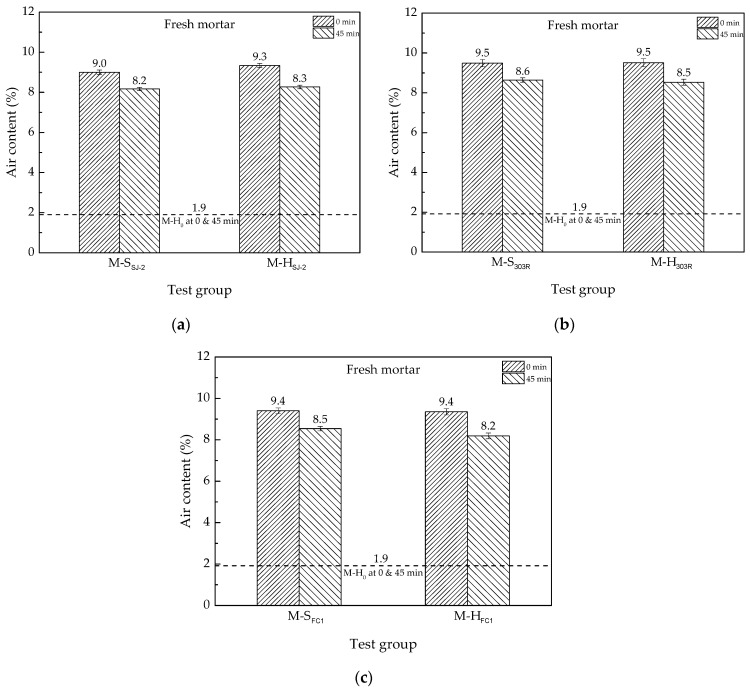
Air contents of fresh mortar prepared in two different atmospheric pressures: (**a**) with SJ-2; (**b**) with 303; and (**c**) with FC1.

In this study, cement/water/sand was constant in both mortar and concrete; therefore, the mortar can be considered as concrete without coarse aggregates. As coarse aggregates have not much air-entraining effect, the air volume in concrete is mainly from its mortar fraction. Therefore, the results of the mortar test can work as an indicator to predict concrete properties. In addition, it is unconfirmable for people to do much physical work at high elevation because the atmosphere there is oxygen-thin. The workload of a batch of mortar test is undoubtedly less than a batch of concrete test, thus the mortar test was available for more reparations to guarantee the accuracy. The air contents of mortar prepared in different atmospheric pressures are shown in Figure 2. Each result is the average of three tests, with 1 L fresh mortar per test.

Figure 2a–c shows a high similarity in air-entraining efficiency between mortar prepared in different atmospheric pressures. With each AEA at the recommended dosage, the initial air content of fresh mortar, tested immediately after mixing, was 9.0–9.5% in either Shigatse or Harbin. This indicates that atmospheric pressure brings little effect on the air-entraining efficiency in the mortar studied. If the test time was 45 min after mixing finished, the air contents of all mortar groups were 8.2–8.6%. According to a Chinese voluntary standard JTG/T D31-06—2017 [23], these mortar specimens were anticipated to survive from 200–400 quick freeze-thaw cycles.

The air content loss in the first 45 min after mixing finished is shown in Table 6.

From Figure 2 and Table 6, it can be found that neither the initial air-entraining efficiency nor the loss rate of air content of fresh mortar, namely the stability of entrained air bubbles, were obviously affected by the atmospheric pressure.

#### 3.2.2. Hardened Mortar

**Figure 3:**
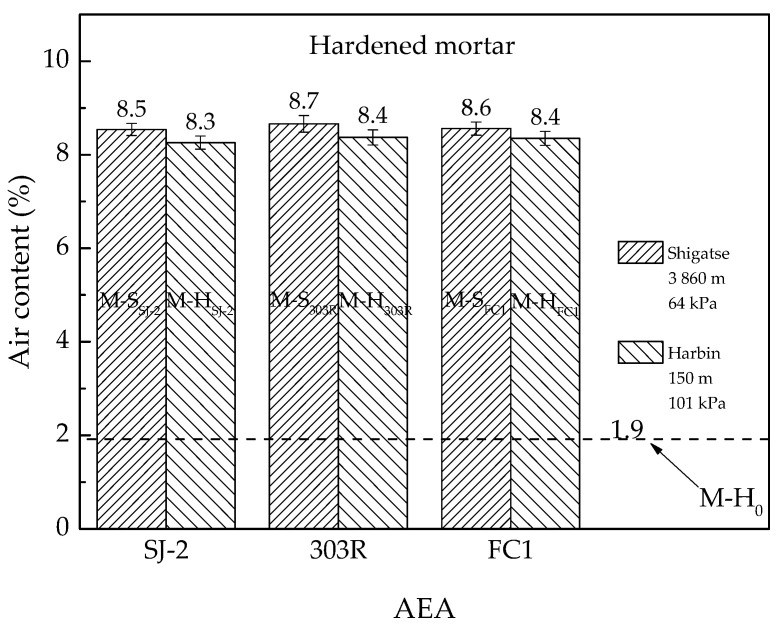
Air contents of hardened mortar prepared in two different atmospheric pressures.

The density of no-air-entrained hardened mortar M-H_0_ in saturated surface dry condition was 2291 kg/m^3^. This value was the average of six mortar cubes, each of which were in the size of 70.7 mm × 70.7 mm × 70.7 mm. Assuming that the air content of M-H_0_ at hardened state was the same as that at fresh state, the air content of M-H_0_ was 1.9%. Thus, the air contents of other groups of hardened mortar could be calculated according to their densities by following Equation (1). The results are shown in Figure 3. Each result is the average of six tests, with one mortar cube per test.

Figure 3 shows that the air content of hardened mortar in Shigatse was slightly higher than that in Harbin. No evidence in mortar tests of this study supports the idea that the air entrainment would be significantly weakened as atmospheric pressure reduces. However, this conclusion is not applicable in a broader context as there are various types of AEAs in the market and this study has only applied three of them.

### 3.3. Air Entrainment in Concrete Prepared in Low Atmosphric Pressure

The air contents of freshly mixed concrete are shown in Table 7. Each result of the air content is the average of three tests. As discussed before, the air content of concrete is mainly from the mortar fraction. According to engineering experience, the volume of mortar fraction is approximately 60% of concrete. Thus, the air content of concrete is anticipated to be about 60% of the mortar with the same water/cement ratio and sand/cement ratio. In Table 7, the air content of 60% of mortar is calculated from M-S (0 min) of Figure 2.

In Figure 2 and Table 7, the air entrainment in concrete was found to be more affected by the type of the used AEA than that in mortar. It could be owing to the impurity substances such as the stone powder and clay in coarse aggregates. These impurity substances have different absorption performances when they contact different types of AEAs, which would influence the air entrainment in concrete. In mortar tests, the aggregates were only clean sands with ISO standard, thus the air entrainment in mortar was little affected. In addition, the uniform degree of coarse aggregates among different batches of concrete might also induce errors.

Although the air content of concrete with FC1 type AEA failed to match 60% air content of mortar with FC1, concrete with SJ-2 and 303R type AEAs presented anticipated air contents. In British standard BS EN 206:2013 [24], the recommended minimum air content for concrete exposed to freeze-thaw attack is 4.0%, while in Chinese standard JTJ 55-2011 [25], the required minimum air content for concrete (nominal maximum size of aggregate ≤25 mm) exposed to freeze-thaw attack is 5.0%. Tests in Lhasa verified that the air content of concrete with either SJ-2 or 303R type AEA satisfied both British and Chinese standards. That is to say, in the region with an atmospheric pressure of 66 kPa, the air-entraining requirement for frost resistance of concrete can still be met by using SJ-2 or 303R type AEA. Therefore, it is not necessary to worry excessively about the freeze-thaw attack resistance of concrete at high elevation if a suitable AEA is appropriately used.

### 3.4. Air Void Chacracteristics of Mortar Prepared in Different Atmosphric Pressures

The air void characteristics of mortar prepared in different atmospheric pressures are shown in Table 8, in which the data are from three cross sections (70.7 mm × 70.7 mm × 3). As the mix proportion of the mortar is the same as the mortar fraction in concrete and Table 7 confirms that the air content of concrete with SJ-2 or 303R type AEA roughly matches to 60% of the air content of the corresponding mortar, the air void characteristics of mortar shown in Table 8 can also be approximately considered as the air void characteristics of concrete with the same AEA.

The air content of hardened mortar presented in Table 8 is not absolutely the same as that presented in Figure 3. The errors might be caused by different test methods. However, in both Figure 3 and Table 8, the air content of hardened mortar prepared in low atmospheric pressure is similar to that prepared in standard atmospheric pressure.

Both the average air void radius and the specific surface area in Table 8 indicate the air void size in hardened mortar. Table 8 shows that air voids in the hardened mortar with 303R type AEA are smaller in low atmospheric pressure, while the size of air voids in the hardened mortar with SJ-2 or FC1 type AEA prepared in low atmospheric pressure is basically equal to that prepared in standard atmospheric pressure. 

The air void spacing factor, which is another important parameter for frost resistance in addition to the air content, was also found to be similar between hardened mortar prepared at different elevations. Therefore, the atmospheric pressure during mixing and hardening would not bring significantly adverse effects on the air void structure or frost resistance of mortar and concrete. 

### 3.5. Significance of Temperature of Raw Materials in Air Entrainment

**Figure 4:**
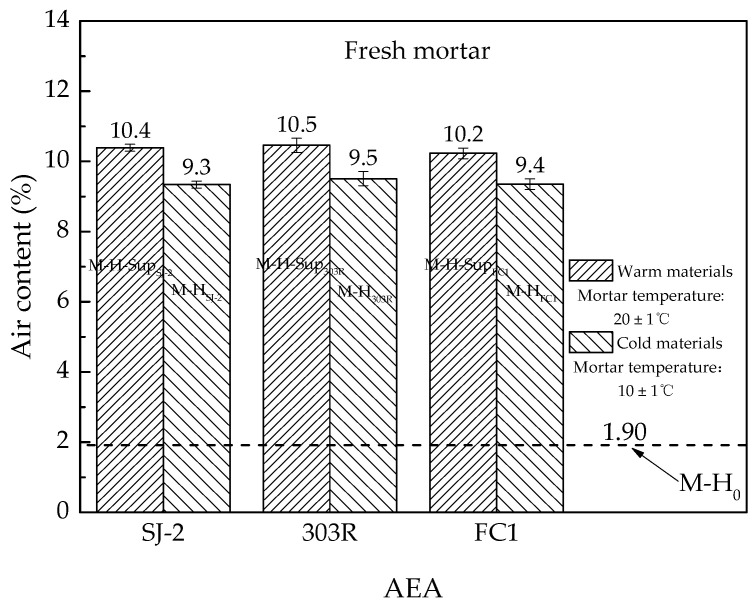
Air contents of fresh mortar made from raw materials with different temperatures.

Figure 4 delivers the results of a supplementary investigation, which pointed out the effect of the temperature of raw materials on the air content of fresh mortar. Each result is the average of six tests, with 1 L fresh mortar per test.

The significance of the temperature of raw materials in air entrainment is clearly shown in Figure 4. After the cooling pretreatment, the air content of mortar met a decrease of about 1.0% (absolute difference), namely 9.5% in percentage. Therefore, it is no wonder that engineers found the air content of concrete at high elevation lower than that at low elevation, because in engineering practice, raw materials at high elevation are cooler than those at low elevation. Hence, the temperature control of raw materials is very important for tests conducted at different elevations, otherwise, the negative effect of the low temperature of raw materials on air entrainment at high elevation might be mistakenly blamed on the low atmospheric pressure.

## 4. Discussion

### 4.1. Significance and Limitations

The study is focused on the potential effect of typical environmental factors at high elevation such as low atmospheric pressure and low temperature on the air entrainment in cement-based materials with several types of commercially-available AEAs.

As concrete tests were only conducted in low atmospheric pressure, instead of both low and standard atmospheric pressures, this study cannot draw a conclusion that the air entrainment of concrete is not affected by atmospheric pressure at all. However, as (i) the air entrainment in mortar is basically unchanged (Figure 2 and Figure 3) when mortar tests are conducted in different atmospheric pressures and (ii) the engineering experience that the air content of concrete is approximately 60% of the air content of mortar is also applicable for several types of AEAs in low atmospheric pressure (Table 7), it is reasonable to conclude that the concrete (with appropriate AEA) prepared in different atmospheric pressures should have similar air contents. More importantly, the study verified that it is easy to entrain enough air content in concrete in low atmospheric pressure regions to satisfy the requirement of freeze-thaw attack resistance. Consequently, although the study cannot rule out that there might be a slight difference in the air entrainment between concrete prepared in different atmospheric pressures, the potential difference has little significance in the sense of practical engineering.

The study helps to correct the preconception of many researchers, who believe that AEAs would perform significantly worse in lower atmospheric pressure [9,12,13,14,15,16]. Indubitably, there are many factors of the AEA applied that would affect the air-entraining efficiency and air bubble stability, including surface tension, critical micelle concentration, hydrophilicity and hydrophobicity, and so on. The application of different AEAs might lead to different results, from which it is possible to find poorer air entrainment in lower atmospheric pressure, as some previous publications stated. One of the most important contributions of the study is recommending two types of AEAs, which are not sensitive to atmospheric pressure, for concrete engineering in high elevation regions. However, whether other common AEAs are feasible in low atmospheric pressure still needs further investigation.

### 4.2. Comparsion with Other Studies

There are huge differences between the findings of this study and those of some other studies [12,13,14,15,16] based on simulated atmospheric pressure, which are mentioned in the introduction section. However, the methodology of those studies is challenged and discussed in detail in Appendix A. Those studies [12,13,14,15,16] claimed that the air-entraining efficiency of AEAs is much lower in low atmospheric pressure compared with standard atmospheric pressure, but such a phenomenon is not found in this on-site study.

The tests results of SJ-2 type AEA at different elevations are also opposite to those of the earliest on-site study in this topic, which was conducted in Golmud (70 hPa) and Beijing (101 hPa), respectively, by Zhu [9]. Zhu found that the air content in Golumd was only approximately half of that in Beijing (details about raw materials and test methods at different elevations were not provided in Zhu’s paper [9]). However, Li [11] also studied the SJ-2 type AEA in Lhasa (66 kPa) and Beijing (101 kPa), respectively. In Li’s study, the air contents of concrete in Lhasa and Beijing were 6.9% and 7.2%, respectively; the two values were similar to each other. The results of Li [11] are consistent with the finding of this study.

The comparison of air void characteristics of mortar prepared in different atmospheric pressures is similar to the research outcomes of Chen [4]. Both studies agree on the similarity of air void characteristics in different atmospheric pressures, suggesting that some other environmental factors such as low temperature would bring more effects than low atmospheric pressure on the air void structure of concrete.

### 4.3. Other Potential Effect of Low Atmospheric Pressure

Although Ran’s research [15] found that cement-based materials presented higher viscosity at the early stage of mixing in lower air pressure, the study of this paper did not find any particular phenomena during mixing and casting at different elevations. Additionally, slump values of fresh concrete in different atmospheric pressures have been tested by Li [11]; the slump value of concrete with SJ-2 type AEA prepared in low atmospheric pressure was similar with that prepared in standard atmospheric pressure.

Low atmospheric pressure might also affect other properties of cement-based materials. For example, Ge [2] and Zhang [26] found that the strength developments of concrete and mortar cured in low atmospheric pressure were slightly slower than that cured in standard atmospheric pressure owing to the increased rate of water evaporation. However, all specimens in both studies of Ge [2] and Zhang [26] were mixed and set in standard atmospheric pressure. Potential effects induced during mixing and casting in low atmospheric pressure on concrete properties, such as the hydration process and pore structure formation (including gel pores and capillary pores), still need further investigation.

## 5. Conclusions

The study conducted experiments on site at different elevations to investigate the effect of different atmospheric pressures on the air entrainment in cement-based materials. The irrelevant variables, which were in addition to the atmospheric pressure, were controlled rigorously. The conclusions drawn from the study include the following:

– In fresh cement paste, SJ-2 type AEAs perform stably in different atmospheric pressures, however, the air-entraining efficiency of 303R or FC1 type AEA is higher in lower atmospheric pressure.
– The initial air content and the loss rate of air content are similar between fresh mortar prepared in different atmospheric pressures. The tendency is applicable for three types of AEAs studied.
– The air content and the air void characteristics of hardened mortar prepared in different atmospheric pressures are basically unchanged if SJ-2 or 303R is selected as the AEA.
– In low atmospheric pressure, there is no challenge for SJ-2 or 303R type AEA to entrain enough air content in fresh concrete to satisfy the requirements of freeze-thaw attack resistance.
– The low temperature of raw materials stored at high elevation negatively affects the air entrainment in cement-based materials.

These conclusions are very important to correct misunderstandings of people on the effect of atmospheric pressure on the air entrainment in concrete. In the premise that a suitable AEA such as SJ-2 or 303R is applied, it is not necessary to excessively worry about the deterioration of air entrainment at high elevation. However, other potential factors in high elevation regions like the low temperature of raw materials should still be taken seriously.

## Figures and Tables

**Table 1:** Natural environment of test sites.

Site	Altitude, m	Atmospheric Pressure, kPa	Mean Daily Maximum Temperature of December, °C	Mean Daily Minimum Temperature of December, °C
Shigatse ^1^	3860	64	9	−12
Harbin	150	101	−11	−21
Lhasa ^2^	3646	66	8	−8

^1,2^ In Lhasa and Shigatse, cement and aggregates were stored outdoors.

**Table 2:** Temperature conditions of laboratories.

Site	Heating System	Indoor Temperature, °C	Tap Water Temperature, °C
Shigatse	Electric air conditioner	5	4
Harbin ^1^	Water heating	20	18
Lhasa	None	5	8

^1^ In Harbin, cement and aggregates were stored indoors.

**Table 3:** Air-entraining agent (AEA) information.

Item	SJ-2	303R	FC1
Manufacturer	Shanghai Fengyang Industrial Co., Ltd.	Research Institute of Highway, Ministry of Transport	Guiling Chemical Co., Ltd.
Physical state	Solid/powder	Fluid/solution	Fluid/solution
Chemical composition	Triterpenoid saponin	Alkyl polyglycoside	Perfluoroalkyl polyoxyethylene ether
Dosage used	1.63/10^4^	2.70/10^4^	0.34/10^4^

**Table 4:** Pretreatments of raw materials.

Site	Cement and Aggregates	Tap Water	Temperature of Fresh Mixtures, °C
Shigatse	-	Heated to 20 °C	10 ± 1
Harbin ^1^	Moved outdoors for 1 hour	Cooled to 8 °C	10 ± 1
Lhasa	-	-	10 ± 1

^1^ If raw materials did not experience the pretreatment, the temperature of fresh mortar mixed in Harbin was 20 ± 1 °C.

**Table 5:** Mix proportions of cement-based materials.

Specimen	Test Type	Test Site	AEA	Cement/Water/Sand/Pebble
P-S_SJ-2_	Cement paste	Shigatse	SJ-2	1:0.45:0:0 (1450 g per batch)
P-S_303R_			303R	
P-S_FC1_			FC1	
P-H_0_		Harbin	-	
P-H_SJ-2_			SJ-2	
P-H_303R_			303R	
P-H_FC1_			FC1	
M-S_SJ-2_	Mortar	Shigatse	SJ-2	1:0.45:2:0 (2329 g per batch)
M-S_303R_			303R	
M-S_FC1_			FC1	
M-H_0_		Harbin	-	
M-H_SJ-2_			SJ-2	
M-H_303R_			303R	
M-H_FC1_			FC1	
M-H-Sup_SJ-2_^1^			SJ-2	
M-H-Sup_303R_			303R	
M-H-Sup_FC1_			FC1	
C-L_0_	Concrete	Lhasa	-	1:0.45:2:2.7 (33.22 kg per batch)
C-L_SJ-2_			SJ-2	
C-L_303R_			303R	
C-L_FC1_			FC1	

^1^ M-H-Sup is the mortar for the supplementary study mentioned in Section 2.2.2.

**Table 6:** Air content loss ^1^ of mortar prepared in different atmospheric pressures, %.

Mortar with SJ-2		Mortar with 303R		Mortar with FC1	
Shigatse	Harbin	Shigatse	Harbin	Shigatse	Harbin
0.8	1.0	0.9	1.0	0.9	1.1

^1^ The air content loss in the table is expressed as the absolute value of the air content difference instead of a percentage of the difference to initial air content.

**Table 7:** Air contents of concrete prepared in low atmospheric pressure (in Lhasa), %.

AEA	Comparison	Air Content	Deviation ^1^	Is the Air Content of Concrete Anticipated?
Blank	60% of mortar	1.1	+0.2	Yes
	concrete	1.3		
SJ-2	60% of mortar	5.4	+0.3	Yes
	concrete	5.7		
303R	60% of mortar	5.7	+0.6	Yes
	concrete	6.3		
FC1	60% of mortar	5.2	−1.2	No
	concrete	4.0		

^1^ It is the deviation between the air content obtained from concrete and that from the 60% of mortar. The acceptable range of the deviation is ±20% of the air content of concrete with no air entrainment or ±10% of the air content of concrete with air entrainment.

**Table 8:** Air void characteristics of mortar prepared in different atmospheric pressures.

Air Void Characteristic	M-S_SJ-2_	M-H_SJ-2_	M-S_303R_	M-H_303R_	M-S_FC1_	M-H_FC1_
Air content, %	8.9	9.2	8.9	9.1	9.0	9.3
Average air void radius, mm	0.120	0.124	0.125	0.142	0.113	0.110
Specific surface area, mm^2^/mm^3^	25.1	24.3	24.1	21.2	26.6	27.2
Air void spacing factor, mm	0.188	0.190	0.195	0.218	0.177	0.169

## Appendix A. Critical Discussion about Research Outcomes Based on Simulated Air Pressures

Many researchers [12,13,14,15,16] mixed concrete in a sealed environment with simulated air pressures to investigate the effect of low air pressure on the air-entrainment in concrete, including air content and air void characteristics. To date, all the published literatures [12,13,14,15,16] based on this methodology stated that the fresh concrete in their studies was mixed in the simulated low air pressure, but the air content of fresh concrete was tested in the standard atmospheric pressure. This methodology should be challenged.

According to the Boyle-Mariotte’s law, air bubbles entrained in low air pressure during mixing would be inevitably compressed once the fresh concrete is exposed to the standard atmospheric pressure For example, if there is a batch of fresh concrete mixed in 60 kPa, its air content would inevitably reduce when it is exposed to standard atmospheric pressure, which is 101 kPa. The ratio of air content after exposure (*A*_60__→101_) to the original air content before exposure (*A*_60_) can be calculated according to the Boyle-Mariotte’s law as follows:

(A1) $$60\;\mathrm{kPa}\times A_{60}=101\;\mathrm{hPa}\times A_{60\to 101}\;$$

(A2) $$\frac{A_{60\to 101}}{A_{60}}=\frac{60\;\mathrm{kPa}}{101\;\mathrm{kPa}}=0.59$$

Therefore, the test results of air content of fresh concrete mixed in low air pressure should be much lower than the real values.

Li [12,13,14] is the first researcher who applied this methodology. Some test results cited from the most influential paper of Li [12] are listed in the second and third columns of Table A1, while the reanalysis of these results is shown in the last column.

**Table A1:** Tests results cited from Li’s research and a reanalysis [12].

AEA	*A*_60__→101_^1^, %	*A*_101_^2^, %	*A*_60_^3^, %
Abietic soap	2.50	4.60	4.24
Alkyl sulfonate	2.54	4.87	4.31
Saponin	3.03	4.92	5.14
Polyether	2.95	4.92	5.00

^1^*A*_60__→__101_ is the air content of fresh concrete mixed in 60 kPa, but tested in 101 kPa; ^2^ *A*_101_ is the air content of fresh concrete mixed and tested in 101 kPa; ^3^ *A*_60_ is the air content of fresh concrete mixed and tested in 60 kPa, which should be the real value of concrete in the engineering practice in high elevation regions, where the atmospheric pressure is 60 kPa. *A*_60_ can be calculated according to Equation (A2).

The paper of Li [12] claimed that the air-entraining efficiency of all AEAs studied was poorer in lower air pressures. However, from Table A1, it can be found that, after the correction by the Boyle-Mariotte’s law, the conclusion would change significantly. Saponin and polyether type AEAs performed stably in different air pressures, although abietic soap and alkyl sulfonate type AEAs entrained less air in lower air pressures.

Chen [16] also studied the air content of concrete mixed in low air pressures, applying the same methodology as Li [12,13,14]. Chen [16] claimed that the air content (tested in standard atmospheric pressure) would be cut in half when concrete was mixed in 50% standard atmospheric pressure. According to the Boyle-Mariotte’s law, such a huge difference would be mainly owing to the compression of fresh concrete rather than the effect of low air pressure.

In addition, the compression effect discussed might induce errors to the air void structure of concrete. With the compression, isolated air bubbles might contact each other and merge into bigger bubbles, leading to an increase in bubble size and a decrease in bubble numbers.

Consequently, it is not feasible to mix concrete in a simulated low air pressure, but subsequently test the fresh concrete in standard atmospheric pressure. Only when the fresh concrete is kept in the simulated low air pressure till it becomes hardened, can the test results become compelling.
